# Erythrocyte-microglia crosstalk contributing to sex differences in pediatric brain tumorigenesis

**DOI:** 10.21203/rs.3.rs-6323329/v1

**Published:** 2025-12-10

**Authors:** Baoli Hu

**Affiliations:** University of Pittsburgh School of Medicine

## Abstract

Pediatric brain tumors are a leading cause of cancer-related morbidity and mortality, with limited treatment options and a male predominance across all ages of children and adolescents^1,2^. However, the origins and underlying mechanisms of these sex differences in tumorigenesis remain elusive. Here, we use medulloblastoma (MB), the most common malignant brain tumor in children^3,4^, as a model to demonstrate that sex-specific cell and organ interactions slow neural progenitor cell (NPC) differentiation, contributing to a higher incidence and poorer prognosis of MB in males. Single-cell transcriptome analyses of developing human and mouse cerebella, along with *in vitro* and *in vivo* validations, uncover intrinsic sexual dimorphisms in NPCs, regulated by microglia-mediated erythrophagocytosis and lipid transfer. In males, lower Glycophorin A (GYPA) expression in the erythrocyte membrane leads to increased cell damage and elevated microglial erythrophagocytosis. Male microglia also exhibit higher expression of Biliverdin Reductase B (BLVRB) and lower expression of ATP Binding Cassette 1 (ABCA1), which orchestrate bilirubin-lipid metabolism and reduce lipid transfer efficiency from microglia to NPCs, thereby slowing NPC differentiation and maturation in males compared to females. These sexual dimorphisms increase NPC vulnerabilities to oncogenic malignant transformation, contributing to the higher prevalence and metastasis of MB in males. Meta-analyses of clinical data reveal that neonatal hyperbilirubinemia (jaundice) caused by high levels of bilirubin, increases pediatric brain tumor risk. Collectively, these findings highlight the origins of sex differences in neurodevelopment and brain tumorigenesis, offering insights into early screening and prevention of pediatric brain tumors through targeted interventions addressing modifiable risk factors.

## Main

Sex-specific brain development is closely linked to physiological neurodevelopment and neurological diseases, yet it remains highly underexplored^5,6^. Subtle differences in gene expression, cellular plasticity, or extracellular factors related to sexual dimorphism during critical and sensitive periods can significantly impact the brain developmental trajectory, potentially leading to neurodevelopmental impairments, including brain tumors^7,8^. Pediatric brain tumors are closely linked to early deviation in brain development and exhibit sexual dimorphic features, including gene expression profiling, tumor incidences, and prognosis^1,9,10^. Understanding how such differences create a sex bias in brain development, and how this bias contributes to the dimorphic nature of brain tumors, could help identify at-risk populations, guide prevention strategies, and inform treatment preferences.

Here, we focus on medulloblastoma (MB), the most common embryonal brain tumor, as a representative pediatric brain tumor to study sexual dimorphism. Arising in the cerebellum, MB accounts for 15–20% of pediatric brain tumors and 70% of all embryonal brain tumors in children^11–13^. We reveal that sex differences in hematopoietic lineages—specifically erythrocytes and microglia—result in slower cerebellar development in males through bilirubin–ABCA1–mediated lipid transfer. This mechanism underpins the higher prevalence and increased malignancy of MB in males compared to females. The discovery that bilirubin, a byproduct of red blood cell (RBC) breakdown, is a risk factor for pediatric brain tumors, offers valuable insights for potential early prevention strategies.

### Sex differences in tumor incidence and prognosis

**Fig. 1:**
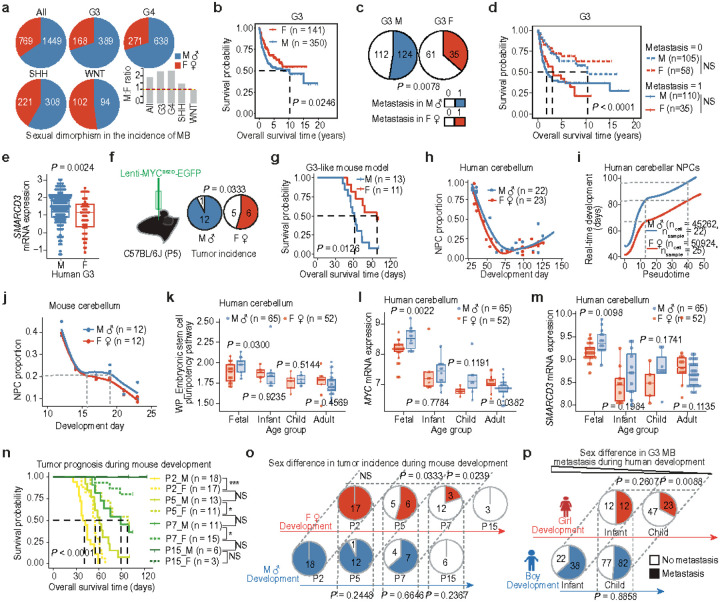
Sex dimorphism in MB incidence, prognosis, and cerebellar NPC development **a**, Pie charts depicting the sex-specific incidence of human MB and bar graphs illustrating the male-to-female incidence ratio across all MB cases and subgroups. **b**, Kaplan-Meier survival curve comparing sex differences in G3 MBs. **c**, Pie charts showing sex-specific incidence of human G3 metastasis. **d**, Kaplan-Meier survival curve of G3 patients stratified by sex and metastasis. **e**, Boxplot showing SMARCD3 expression levels in human G3 between sexes. **f**, Left: schematic diagram illustrating the method of G3 MB induction in mice. Right pie charts showing sex-specific incidence of G3 MB in P5 mice. **g**, Kaplan-Meier survival curve of P5-induced mouse G3 tumors. **h**, Cerebellar NPC proportion across human developmental timelines based on scRNA-seq data. **i**, Pseudotime analysis showing differences in developmental timing between sexes. **j**, Cerebellar NPC proportions across mouse developmental timelines based on scRNA-seq data. **k**, Boxplot showing pathway enrichment scores in the cerebellum across age groups. Boxplot showing expression levels of MYC (**l**) and SMARCD3 (**m**) in the cerebellum across age groups. **n**, Kaplan-Meier survival curve of G3-induced mice stratified by developmental stage and sex. **o**, Pie charts showing sex-specific incidence of G3 in mice induced at different developmental stages. **p**, Pie charts showing sex-specific incidence of human G3 metastases across age groups. Each dot represents one bulk sample (**e**, **k**, **l**, **m**) or one merged sample from scRNA-seq data (**h, j**). n denotes the number of human samples (**b**, **d**, **h**, **i**, **k**, **l**, **m**) or mouse samples (**g**, **j**, **n**), or cells (**i**). Data are presented as the mean ± s.d. (**e**, **k**, **l**, **m**). *P* values were calculated using the log-rank test (**b**, **d**, **g**, **n**), two-tailed Welch’s *t*-test (**e**, **k**, **l**, **m**), or chi-square test (**o**, **p**). ***P = 0.0018 (P2_M *vs*. P2_F), *P = 0.0126 (P5_M *vs*. P5_F), *P = 0.0242 (P7_M *vs*. P7_F), NS, not significant.

We comprehensively analyzed 2,218 MB cases and found that sex is a significant factor influencing tumor incidence, with boys generally experiencing a higher risk of developing MB compared to girls (Fig. 1a and **Extended Data Fig. 1a-d**). Molecular characterization of MB reveals four major subgroups: WNT, SHH, Group 3, and Group 4 (hereafter G3 and G4)^14,15^. Our analysis further demonstrates that G3 MBs exhibit significant sex differences in prevalence and prognosis among the four MB subgroups (Fig. 1a, b and **Extended Data Fig. 1d, e**). As the most aggressive subgroup, nearly 50% of G3 MBs have metastasis at the time of diagnosis, making this the subtype with the worst outcomes^4^. Our analysis reveals that metastasis is more common in males than females with G3 MBs (Fig. 1c and **Extended Data Fig. 1f**), contributing to the poorer prognosis observed in males (Fig. 1d and **Extended Data Fig. 1g**). In our previous study, SMARCD3 (also known as BAF60C) was identified as a key gene highly expressed in G3 MBs, promoting metastasis^16^. We found that SMARCD3 expression levels are significantly higher in male than in female patients with G3 MB (Fig. 1e). Importantly, we identified that the survival difference is not due to the extent of resection, except in cases where the subtotal resection leaves 1.5 cm^2^ or more of residual tumor (**Extended Data Fig. 1h**). These data suggest that elevated SMARCD3 expression in males drives increased metastasis, ultimately contributing to the poorer prognosis of MB patients observed in males compared to females.

To better understand the sex bias driving tumor prevalence and prognosis, we developed an immune-competent C57BL/6J mouse model of G3-like MB by intracerebellar injection of lentivirus overexpressing a phosphomimetic c-MYC S62D mutant (pLenti-MYC^S62D^), which replicates the high frequency of serine 62 phosphorylation observed in human G3 MBs^17^ (Fig. 1f and **Extended Data Fig. 2a-f**). Consistent with human data, male mice exhibit a higher incidence of tumors, and a poorer prognosis compared with female mice when tumors were induced by pLenti-MYC^S62D^ in developing mice on postnatal day 5 (P5) (Fig. 1f, g). Single-cell RNA sequencing (scRNA-seq) reveals a migrating tumor cell population with elevated SMARCD3 expression in males, a finding that is less pronounced in females (**Extended Data Fig. 2g, h**). These observations align with our human data and highlight the potential role of genetic/epigenetic alterations in the developing cerebellum in driving sex differences in MB formation and prognosis.

### Sexual bimaturism in NPCs and MB

To delineate whether early cerebellar development contributes to sexual dimorphism in tumorigenesis, we first analyzed gene expression profiling data from MB patients and found a significant enrichment of the G3 tumor signature in neural progenitor cells (NPCs), indicating NPCs as the cell-of-origin for G3 MB (**Extended Data Fig. 3a**), which is supported by other scRNA-seq based studies^18–20^. Analysis of scRNA-seq data from developing human and mouse cerebella reveals slower NPC maturation rates in males compared to females, along with neurodevelopment and potential MB development signatures enriched in sex-differentially expressed genes (DEGs) (Fig. 1h–j and **Extended Data Fig. 3b-g**). This observation is reinforced by bulk RNA-seq data analyses, which show increased activity in the embryonic stem cell pluripotency pathway and elevated MYC and SMARCD3 expression levels in male cerebella during the fetal stage, but not in childhood or adulthood (Fig. 1k–m and **Extended Data Fig. 3h**). These findings suggest that slower NPC differentiation and maturation during this critical early developmental period in males enhances tumor formation potential and extends the risk window, thereby increasing susceptibility to MYC-driven tumorigenesis while promoting metastasis through SMARCD3 expression.

Notably, we observed a significant decrease in tumor incidence and improved progression as the cerebellum matures in our MYC^S62D^-induced G3 mouse MB models (**Extended Data Fig. 4a, b**). Neonates are generally considered to have an immature and developing immune system, making them more vulnerable to pathogenic insults^21^, evidenced by the lower immune cell populations, primarily microglia, during early brain development (**Extended Data Fig. 4c, d**). To evaluate the impact of immune response on tumor incidence and prognosis in early developing mice, cerebellar cells from P2 C57BL/6J mice infected with pLenti-MYC^S62D^ were intracranially implanted into P30 C57BL/6J mice. We found that all P30 recipient mice developed G3-like tumors, with survival rates comparable to those of P2 mice, indicating that the immune system does not significantly impact tumor formation or survival (**Extended Data Fig. 4d, e**). Sex- and age-specific human MB-derived xenograft models show correspondingly distinct survival and metastasis rates in immunodeficient mice^16^, further supporting the role of NPC characteristics over immune factors in influencing tumorigenesis (**Extended Data Fig. 4f, g**). Collectively, the greater abundance and immaturity of NPCs in males contribute to increased tumorigenesis and a higher likelihood of metastatic tumors.

The pool of NPCs is known to decrease significantly as development progresses, regulated by their proliferation, differentiation, and apoptosis^22^. When comparing mouse sex differences in tumor incidence and prognosis, we observed that younger females had a similarly low tumor development potential as males 2–3 days older but a lower potential than age-matched males. Prognosis followed a similar trend: tumors induced in younger females had survival outcomes comparable to males 2–3 days older yet demonstrated 30 days better survival outcomes than age-matched males (Fig. 1n, o). These data suggest that female cerebellar development is approximately 2–3 days ahead of male cerebellar development between P0 and P7 in mice. The observed 2–3-day sex differences in NPC-related stemness during the critical developmental period account for approximately 30 days of survival variance in mice undergoing MB tumorigenesis. Pseudotime analyses of developmental periods in human cerebellum scRNA-seq datasets reveal that female cerebellar development is approximately 20 days ahead of male cerebellum development (Fig. 1i). Under tumorigenesis, the same prevalence of G3 MB occurs in younger females compared to males, *e.g*., a 5.5-year difference: 6 years old in females *vs*. 11 years old in males (**Extended Data Fig. 1d**); Additionally, infant females exhibit metastasis rates similar to those of child males, but much lower than those of infant males (Fig. 1p). These data suggest that the observed 20-day developmental advantage in female NPCs during the critical developmental period accounts for approximately 5.5 years of prevalence variance in human undergoing MB tumorigenesis. Collectively, our mouse and human data demonstrate subtle yet significant sexually dimorphic cerebellar development, with these sex differences profoundly influencing MB incidence and prognosis.

### Microglia-NPC interaction in MB tumorigenesis

**Fig. 2:**
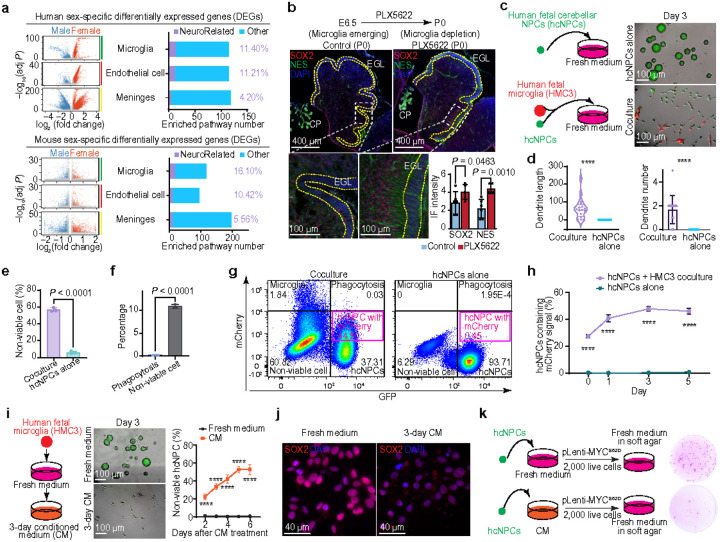
Microglia promote NPC differentiation through secreted factors. **a,** Volcano plot illustrating the sex-specific DEGs in microenvironment cells. Bar graphs showing the percentage of neurodevelopment related pathways among GO-enriched DEGs. **b**, Schematic diagram of the experimental procedure (top). IF images of the cerebellum and quantification of marker gene expression in control *vs*. PLX5622-treated mice at PO. White dashed line boxed indicate regions shown in the lower panels. **c**, Schematic diagram of the experimental procedure (left); bright-field and fluorescence images of cocultured cells (right). **d**, Violin plot showing dendrite length of each hcNPC (left); boxplot depicting the number of dendrites of each hcNPC (right). **e**, Histograms showing the percentage of non-viable hcNPCs. **f**, Histograms showing the percentage of phagocytic and non-viable hcNPCs during coculture. **g**, Flow cytometry analysis comparing separate cultures *vs*. cocultured mCherry-labeled microglia and GFP-labeled hcNPCs. **h**, Percentage of hcNPCs containing mCherry signal at different coculture time points. **i**, Schematic diagram of the experimental procedure (left), bright-field and fluorescence image (middle) and quantification of non-viable cell percentages of hcNPCs cultured in fresh *vs*. conditioned medium (right). **j**, IF images of hcNPCs cultured in fresh *vs*. conditioned medium. **k**, Schematic diagram of the experimental procedure (left) and soft agar assay images comparing hcNPCs cultured in fresh *vs*. conditioned medium (right). Each dot represents a biologically independent sample (**b**, **e**, **f**) or an individual cell (**d**). Data are presented as the mean ± s.d. (**b**, **d**, **e**, **f**, **h**, **i**). *P* values were calculated using one-tailed unpaired *t*-test (**b**) or two-tailed unpaired *t*-test (**d**, **e**, **f**, **h**, **i**). ****P < 0.0001. EGL, external granule layer; CP, choroid plexus.

Given that the sex differences in MB tumorigenesis are mainly in prepubertal children, where circulating sex hormones are at their lifetime nadir^23^, we focus on how the developing cerebellar microenvironment, an emerging key player in brain tumorigenesis^24^, influences sexual dimorphism and vulnerabilities of NPCs during tumorigenesis. By integrating human and mouse scRNA-seq data to compare the signatures of sex-related DEGs across major cell types interacting with NPCs, including microglia, endothelial cells, and meninges within the early brain milieu^25–27^, we found that microglia emerge as a critical non-neural lineage cell type, with their sex-related DEGs showing more enrichment in neurodevelopmental pathways compared to those in other two cell types (Fig. 2a). This finding is further supported by spatial transcriptomic analyses and immunofluorescence staining (IF), which demonstrate the close physical proximity of microglia and NPCs during early development (**Extended Data Fig. 5a-d**). Pharmacological depletion of microglia in the developing mouse brain enhances NPC stemness, evidenced by significant increases in the expression levels of SOX2 and Nestin, along with a reduction in GFAP, MAP2, and TUJ1 expression in the cerebellum (Fig. 2b and **Extended Data Fig. 5e-g**). Moreover, MYC and SMARCD3, key drivers in MB tumorigenesis^16^, are highly expressed in the mouse cerebella following microglia depletion (**Extended Data Fig. 5h, i**). Strikingly, we also observed cerebellar heterotopias in the nodulus of microglia-depleted mice (**Extended Data Fig. 5j**), which are considered premalignant lesions for G3 and G4 MBs^28^. In contrast, co-culturing human fetal cerebellar NPCs (hcNPCs)^16,29^ with human fetal microglia (HMC3)^30,31^ cells induces NPC differentiation, characterized by the development of long dendrites, and ultimately promotes cell death (Fig. 2c–e and **Extended Data Fig. 6a-c**). NPC death was initially thought to result from microglial phagocytosis, one of the most ancient and fundamental mechanisms of cell death. Surprisingly, we found phagocytosis only accounted for 0.2% of cell death in the developing cerebellum, based on imaging and flow cytometry analyses of co-cultures of hcNPCs with human fetal microglia, along with scRNA-seq data analysis of human and mouse cerebella (Fig. 2f and **Extended Data Fig. 6d-f**). We also observed that approximately half of hcNPCs (45 ± 4.89%) in coculture contain microglial secreted factors (Fig. 2g, h and **Extended Data Fig. 6d**), indicating that microglia primarily promote NPC differentiation through secreted factors. Additional evidence shows that hcNPCs treated with human fetal microglial conditioned medium (CM) exhibit induced differentiation, mirroring the effects observed in direct co-culture with microglia (Fig. 2i, j and **Extended Data Fig. 6g-j**). Furthermore, hcNPCs treated with the CM lost their tumorigenic capacity, as shown in a soft agar assay (Fig. 2k). These findings suggest that NPC differentiation and tumor formation inhibition are mediated by microglia-secreted factors (**Extended Data Fig. 6k**).

**Fig. 3:**
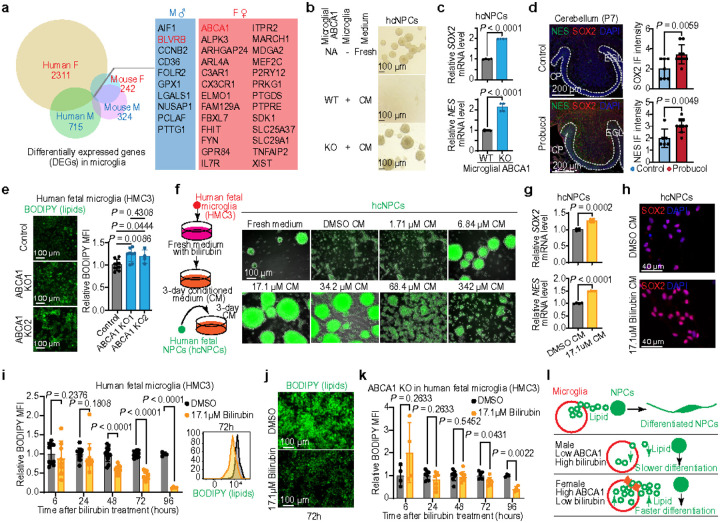
Sex-dimorphic bilirubin-ABCA1 mediated microglial lipid transfer regulates NPC differentiation paces. **a,** Venn diagram showing overlapping DEGs in microglia between males and females across human and mouse datasets. **b**, Bright-field image of hcNPCs cultured in the indicated conditioned media. **c**, RT-qPCR analysis of SOX2 and NES mRNA expression in hcNPCs cultured in wildtype (WT) microglia-conditioned medium *vs*. ABCA1 KO microglia-conditioned medium. **d**, IF images of the cerebellum and quantification of indicated marker genes in control *vs*. probucol-treated mice. **e**, Fluorescence images (left) and How cytometry analysis (right) of median fluorescence intensity (MFI) of BODIPY-stained microglia in control *vs*. ABCA1 KO conditions. **f**, Schematic diagram of the experimental procedure for culturing hcNPCs using bilirubin-treated microglia-conditioned media (left); bright-field and fluorescence images of hcNPCs cultured in conditioned media from microglia treated with different bilirubin concentrations (right). **g**, RT-qPCR analysis of SOX2 and NES mRNA expression in hcNPCs cultured in the conditioned media from DMSO- *vs*. bilirubin-treated microglia. **h**, IF images of hcNPCs cultured in conditioned media from DMSO- *vs*. bilirubin-treated microglia. **i**, MFI of BODIPY in microglia treated with DMSO vs. bilirubin for different durations. **j**, Fluorescence images of BODIPY-stained microglia treated with DMSO *vs*. bilirubin for 72 hours. **k**, MFI of BODIPY in ABCA1 KO microglia treated with DMSO *vs*. bilirubin for different durations. **l**, Schematic diagram illustrating how reduced lipid transfer due to low ABCA1 expression and increased lipid consumption driven by high bilirubin levels contribute to slower NPC differentiation in males. Each dot represents a biologically independent sample (**c**, **d**, **e**, **g**, **i**, **k**). Data are presented as the mean ± s.d. (**c**, **d**, **e**, **g**, **i**, **k**). *P* values were calculated using two-tailed unpaired *t*-test (**c**, **d**, **g**, **i**, **k**) or one-way ANOVA with FDR correction (**e**).

To determine whether there are sex-specific microglial secretomes in males and females, we compared the DEG profiles of human and mouse microglia. These sex-specific DEGs are significantly enriched in cellular secretion processes and membrane-bound pathways, highlighting the critical role of microglial secretions in cerebellar development and sex differences (Fig. 3a and **Extended Data Fig. 7a-d**). To identify the secretory genes contributing to sex-specific microglial functions that influence NPC differentiation, we performed CRISPR-based screening on the DEGs. This analysis reveals ATP binding cassette 1 (ABCA1), which is highly expressed in female microglia compared to male microglia, as a key regulator of NPC differentiation *in vitro* (Fig. 3b, c and **Extended Data Fig. 8a-d**). *In vivo* pharmacological inhibition of ABCA1 function further verifies its role in promoting cerebellar NPC differentiation (Fig. 3d and **Extended Data Fig. 8e-h**). These findings indicate that higher expression of ABCA1 in female microglia enhances the differentiation and maturation of NPCs more effectively than in male microglia.

### Fine-tuning sexual dimorphism in NPC differentiation

ABCA1 is a member of the ATP-binding cassette superfamily of transporters, responsible for facilitating cellular lipid efflux and maintaining lipid homeostasis in the brain^32,33^. To further explore whether ABCA1 and its network-regulated lipid metabolism contribute to sex differences in microglia-mediated NPC differentiation, BODIPY and CholEsteryl BODIPY (BODIPY^CE^) lipid transfer assays were performed to screen the sex-specific DEGs in microglial secretomes. We found that CRISPR-mediated knockout (KO) of the *ABCA1* gene in microglial HMC3 cells led to significant intracellular lipid accumulation and a reduction in lipid transfer from microglia to hcNPCs (Fig. 3e and **Extended Data Fig. 9a-d**). In contrast, the Biliverdin Reductase B (BLVRB) KO significantly enhances lipid transfer from microglia to hcNPCs, promoting hcNPC differentiation (**Extended Data Fig. 9b-e**). BLVRB, known to reduce biliverdin to bilirubin, plays a predominant role during early development^34,35^. We found that BLVRB is highly expressed in male microglia compared to female microglia (Fig. 3a and **Extended Data Fig. 9f, g**), possibly explaining the previously observed higher bilirubin levels in male newborns^36,37^. To further test our hypothesis that bilirubin influences microglia-mediated NPC differentiation, we treated HMC3 microglial cells with varying bilirubin concentrations, and the microglial CM were collected to culture hcNPCs (Fig. 3f). The results show that hcNPC differentiation is significantly inhibited when exposed to microglial CM at concentrations from 6.84 μM to 34.2 μM, including 17.1 μM, which corresponds to the normal maximum level in human serum and in the cerebellum of the hyperbilirubinemic Gunn rat model^38,39^; however, notable cell death was observed at higher concentrations of 68.4 μM and 342 μM (Fig. 3f–h and **Extended Data Fig. 9h, i**). These results indicate that the BLVRB/bilirubin cycle plays a crucial role in microglial regulation of sex-dimorphic neurodevelopment.

While bilirubin, a component of the heme catabolic pathway, is essential for liver, spleen, and bile functions^34,40^, the role of the BLVRB/bilirubin cycle in microglial metabolism remains largely unknown. To investigate whether bilirubin directly regulates microglial lipid metabolism, we treated human fetal microglial cells with bilirubin and observed a decrease in intracellular lipid levels after 48 hours of exposure (Fig. 3i, j). Since ABCA1 deletion impairs lipid efflux, leading to intracellular lipid accumulation in microglia (Fig. 3e), we hypothesized that bilirubin treatment would not reduce intracellular lipid levels in ABCA1-deficient microglia if BLVRB/bilirubin relies on the ABCA1-mediated lipid transfer pathway.

However, we observed that bilirubin reduces the intracellular lipid accumulation in ABCA1-deficient HMC3 cells with a delayed reduction in lipid levels occurring at 72 hours in ABCA1-deficient cells compared to 48 hours in wildtype cells (Fig. 3k). This finding suggests that bilirubin reduces lipid levels through a mechanism independent of ABCA1-mediated lipid transfer, possibly by binding to PPARα to inhibit lipid accumulation^41^. Our integrated analysis of human and mouse scRNA-seq datasets reveals that ABCA1 is lowly expressed, while BLVRB is highly expressed in developing male microglia compared to female microglia (**Extended Data Fig. 8b-d** and **9f-g**). Collectively, these findings suggest that sex differences in microglia-mediated NPC differentiation during early brain development are fine-tuned through two distinct lipid mechanisms: in males, higher BLVRB/bilirubin levels maintain lower lipid storage, while lower levels of ABCA1 further limit cellular lipid/cholesterol efflux in male microglia. These coordinated processes lead to delayed NPC differentiation, slowed cerebellar maturation, and increased NPC vulnerabilities to tumorigenesis during cerebellar development, contributing to the higher prevalence and metastasis of MB in males compared to females (Fig. 3l).

### Sexual dimorphism of microglial erythrophagocytosis

**Fig. 4:**
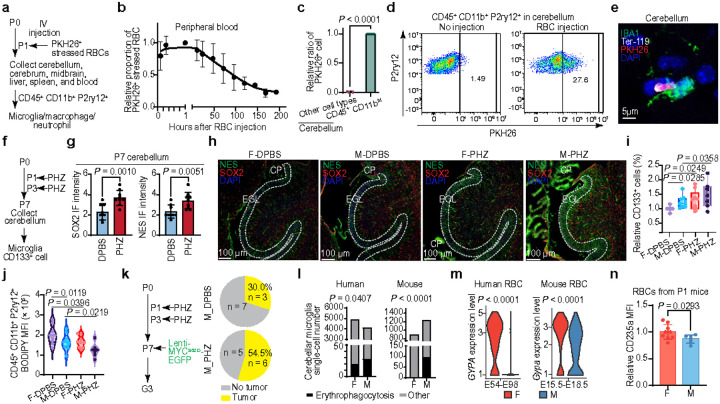
Fragile erythrocytes and enhanced cerebellar microglial erythrophagocytosis elevate MB incidence. **a**, Schematic diagram illustrating the procedure for monitoring erythrophagocytosis activity. **b**, Dot plot showing the proportion of peripheral injected erythrocytes over time. **c**, Histogram depicting the relative proportion of cerebellar erythrophagocytic cells. **d**, Flow cytometry analysis of erythrophagocytosis in cerebellar CD45^+^ CD11b^+^P2ry12^+^ cells. **e**, IF image showing erythrophagocytosis in the P7 mouse cerebellum. **f**, Schematic diagram outlining the flow cytometry procedure following PHZ treatment. **g**, Quantification of indicated marker genes by IF staining in control *vs*. P HZ-treated mice. **h**, Cerebellar IF images of mice treated with DPBS or PHZ. **i**, Box plot displaying the relative proportion of CD 133^+^ cells in P7 mice treated with DPBS or PHZ. **j**, Violin plot showing BODIPY MFI in cerebellar microglia from P7 mice treated with DPBS or PHZ. **k**, Schematic diagram (left) showing the procedure, and pie chart (right) depicting the proportion of tumor induction following PHZ treatment in mice. **l**, Quantification of erythrophagocytic microglia in the developing cerebellum. **m**, Expression levels of GYPA in human and mouse erythrocytes. **n**, GYPA MFI in P 1 mouse erythrocytes. M, male; F, female (**h**-**n**). Each dot represents one biologically independent sample (**c**, **g**, **i**, **j**, **n**). Data are presented as the mean ± s.d. (**b**, **c**, **g**, **i**, **n**). *P* values were calculated using a one-tailed unpaired *t*-test (**g**, **i**, **j**, **n**), two-tailed unpaired *t*-test (**c**), chi-square test (**l**), or non-parametric Wilcoxon rank-sum test (**m**).

Bilirubin is predominantly produced and released by macrophages in the liver through a process known as erythrophagocytosis, wherein macrophages engulf senescent and damaged erythrocytes^42^. However, the occurrence of microglia mediated erythrophagocytosis in the cerebellum remains elusive. To this end, stressed red blood cells (RBCs) were intravenously delivered to P1 mice (Fig. 4a); the observed decrease in labeled RBCs within peripheral blood indicates effective erythrophagocytosis for RBC clearance (Fig. 4b and **Extended Data Fig. 10a**). Flow cytometry and IF staining analyses further reveal that cerebellar microglia possess erythrophagocytic capabilities in the cerebellum, suggesting that the cerebellar microglia are able to produce bilirubin through erythrophagocytosis (Fig. 4c–e and **Extended Data Fig. 10b**). To determine the impact of microglial erythrophagocytosis on NPC differentiation through the bilirubin-lipid cycle, we generated a phenylhydrazine (PHZ)-induced hemolytic hyperbilirubinemia model by inducing RBC damage and subsequent erythrophagocytosis in C57BL/6J mice^43^ (Fig. 4f). In this model, we observed reduced NPC differentiation, characterized by lower expression of GFAP, TUJ1, and MAP2, along with elevated expression of SOX2, Nestin, and CD133 (Fig. 4g–i; **Extended Data Fig. 11** and **12a-c**). Additionally, we noted a decrease in microglial lipid content and cerebellar deficits, characterized by a reduced number of cerebellar folia in PHZ-treated mice (Fig. 4j and **Extended Data Fig. 12d**). These effects are more pronounced in PHZ-treated males than in females. Importantly, we observed a higher tumor incidence in male PHZ-treated C57BL/6J mice following intracerebellar injections of pLenti-MYC^S62D^-EGFP (Fig. 4k). These findings substantiate our hypothesis that microglial erythrophagocytosis-mediated heme-bilirubin-lipid cascade inhibits NPC differentiation and enhances susceptibility in NPC tumorigenesis.

To determine whether male cerebellar microglia produce higher bilirubin levels under physiological conditions, we examined sex differences in microglial erythrophagocytosis. Analysis of scRNA-seq datasets from human and mouse developing brains reveal significantly greater erythrophagocytic activity in male microglia (Fig. 4l). We did not observe an inherently higher NPC-related phagocytic function in male microglia compared to female microglia (**Extended Data Fig. 6f**). Therefore, we hypothesize that sex differences in erythrocytes may drive the increased erythrophagocytic activity observed in male microglia. Sex-biased gene expression analysis in human and mouse data reveals that Glycophorin A (GYPA) expression levels are lower in male erythrocytes compared to female erythrocytes (Fig. 4m and **Extended Data Fig. 13a**). Our experimental data further confirm lower GYPA protein expression in RBCs from P1 male C57BL/6J mice compared to females (Fig. 4n and **Extended Data Fig. 13b**). GYPA, also known as CD235a, plays a pivotal role in regulating erythrocyte biology, including maintaining mechanical stability and decreasing membrane deformability^44^. Our results suggest lower GYPA levels in male RBCs contribute to a fragile membrane causing more erythrocytes in damage and triggering enhanced microglial erythrophagocytosis activity. Collectively, these results indicate that the low GYPA expression in male RBCs leads to increased heme release from fragile RBCs, subsequently driving the heme-biliverdin-bilirubin cycling, promoting microglial lipid reduction, slowing microglia-mediated NPC differentiation. This cascade ultimately contributes to a higher incidence of hyperbilirubinemia and susceptibility to tumorigenesis in males compared to females.

### Bilirubin and pediatric brain tumor risk

**Fig. 5:**
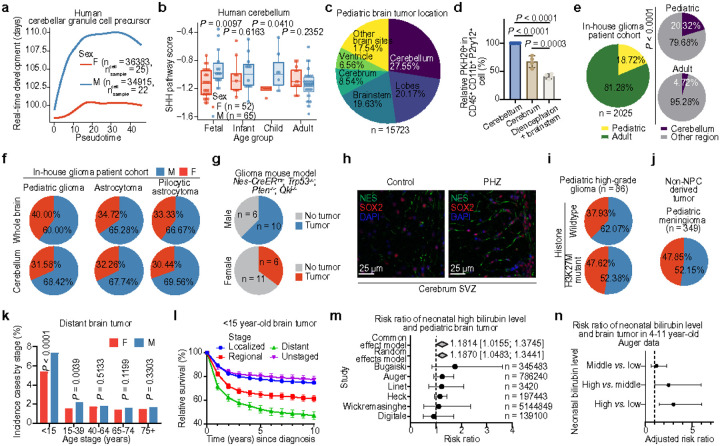
Erythrocyte-microglia-NPC crosstalk links to pediatric brain tumor development **a,** Pseudotime analysis showing developmental timing differences between sexes at the same human GCP state. **b**, Boxplot showing SHH pathway scores in the human cerebellum across age groups. **c**, Pie chart depicting the distribution of pediatric brain tumors by location. **d**, Histogram comparing microglial erythrophagocytosis activity in different brain regions in PKH26 labeled erythrocyte-injected mice. **e**, Pie charts showing the distribution of in-house glioma cases across different groups. **f**, Pie charts illustrating sex differences in the incidence of indicated pediatric glioma subtypes. **g**, Pie chart showing sex differences in a spontaneous mouse glioma model. **h**, IF images of cerebrum SVZ in control and PHZ-treated mice showing indicated marker gene expression. **i**, Pie chart representing sex differences in pediatric high-grade glioma incidence. **j**, Pie chart representing sex differences in pediatric meningioma incidence. **k**, Bar graph showing the percentage of distant tumors in different sexes across age groups. **l**, Relative survival percentages across pediatric brain tumor stages. **m**, Forest plot showing the risk ratio of pediatric brain tumor susceptibility associated with higher bilirubin levels at birth. **n**, Forest plot showing the risk ratio of brain tumors diagnosed between ages 4–11 associated with different bilirubin levels at birth. Each dot represents a biologically independent sample (**b**, **d**). Data are presented as mean ± s.d. (**b**, **d**, **l**) or mean ± 95% confidence intervals (**m**, **n**). *P* values were calculated using a two-tailed unpaired *t*-test (**b**), one-way ANOVA (**d**), or chi-square test (**e**, **k**). SVZ, subventricular zone.

Given the impact of erythrocyte-microglia crosstalk on sex differences in NPC differentiation and tumorigenesis, we hypothesize that similar cellular and molecular mechanisms may contribute to sex differences in other MB subgroups and pediatric brain tumors originating from NPC-related lineages. Granule cell precursors (GCPs) originate from cerebellar NPCs and serve as the cell of origin for SHH MB following the oncogenic activation of Sonic Hedgehog signaling^18,45,46^. Transcriptomic analyses of human GCPs reveal slower differentiation and maturation in male GCPs compared to females, along with the increase of the oncogenic Sonic Hedgehog signaling pathway in males during fetal and childhood stages (Fig. 5a, b). Sex differences in SHH MB incidence are evident, with a male-to-female ratio of 1.4:1 in human datasets (Fig. 1a) and 2.1:1 in a previous SHH mouse MB model^47^. Our mouse models with microglia depletion, ABCA1 inhibitor treatment, and PHZ induced hyperbilirubinemia exhibit a thicker external granule layer, where the GCPs reside, compared to controls (Fig. 2b, Fig. 3d and Fig. 4h). This finding further suggests that GCPs inherit the sex-specific NPC differentiation influenced by the bilirubin–ABCA1-mediated microglial lipid transport system. Clinically, pediatric brain tumors most commonly occur in the cerebellum^1,18,48^, which is further supported by our analysis of 15,723 cases (Fig. 5c). We hypothesize that increased activation of microglial erythrophagocytosis renders the cerebellum more vulnerable to tumorigenesis than other brain regions, following the heme-bilirubin-lipid cycle-driven less differentiated NPC mechanism. Our results reveal that cerebellar microglia possess erythrophagocytosis capabilities comparable to macrophages in the liver and spleen, significantly exceeding those of microglia in the cerebrum and diencephalon and brainstem (Fig. 5d and **Extended Data Fig. 13c-e**). A previous study reported that the cerebellum has the highest bilirubin levels compared to the brainstem and cortex in hyperbilirubinemic Gunn rat pups, with male pups exhibiting significantly higher cerebellar bilirubin levels than their littermate-matched female counterparts^39^. These findings support our hypothesis that elevated cerebellar bilirubin content preferentially predisposes males to the development of pediatric brain tumors.

Besides MB, glioma-particularly low-grade glioma-is the most common pediatric brain tumor (**Extended Data Fig. 13f**). Further analyses of our in-house cohort of 2,025 glioma patients reveal that 20.32% of pediatric gliomas occur in the cerebellum, compared to only 4.72% of adult gliomas (Fig. 5e and **Extended Data Fig. 13g**). A pronounced male prevalence was observed in pediatric gliomas, particularly in pilocytic astrocytoma of the cerebellum, followed by other brain regions (Fig. 5f). To investigate sex differences in NPC-derived glioma development in the cerebrum, we induced tumor formation in a Nestin^+^ cell population-derived mouse glioma model by deleting *Trp53, Pten*, and *Qki* during the early postnatal period^49^. We observed a male-to-female tumor incidence ratio of 1.70:1 occurring in the cortex (Fig. 5g). Additionally, PHZ treatment inhibits NPC differentiation in the cortex subventricular zone, as indicated by increased SOX2 and Nestin expression (Fig. 5h). Children with high-grade pediatric gliomas (pHGGs) exhibit a male prevalence (male: female = 1.64:1) in histone 3 wildtype pHGGs, which exclusively arise in the cerebral cortex, compared to histone 3 K27M mutant pHGGs, which mainly occur in the midline, where the microglia possess less erythrophagocytosis capabilities, especially brainstem (Fig. 5i). Additionally, no significant sex differences in tumor incidence were observed in non-NPC-derived pediatric meningiomas (Fig. 5j). Furthermore, male patients with brain tumors under 15 years of age are more likely to present with distant or metastatic tumors at diagnosis and have poorer prognoses in populations with metastases compared to those without (Fig. 5k, l). Collectively, these findings underscore the critical interaction between NPC and the brain microenvironment/locations in shaping sex differences in pediatric brain tumorigenesis.

In clinical settings, high levels of bilirubin can lead to hyperbilirubinemia (jaundice) and cause neurological damage when bilirubin accumulates in the brain^50^. To determine if higher levels of bilirubin can serve as a risk factor for pediatric brain tumor development, we did meta-analysis based on the published epidemiological datasets^51–56^. Given the lack of direct bilirubin level information in most of these cases, we categorized patients into high- and low-level bilirubin groups based on whether infants received phototherapy, a standard treatment for continuous and severe neonatal hyperbilirubinemia^57^. One dataset further confirms significantly higher total serum bilirubin levels in the phototherapy-treated cohort compared to the untreated group (**Extended Data Fig. 13h**). Our meta-analysis covering 6.6 million pediatric cases reveals that neonatal hyperbilirubinemia elevates the risk of pediatric brain tumors by 1.2-fold in all ages between 0–19 (Fig. 5m). Most children diagnosed with common malignant pediatric brain tumors, such as gliomas and MBs, are between the ages of 4 and 11 (**Extended Data Fig. 13i**). Considering the two-hit hypothesis and the incubation period of bilirubin ramifications, we focus on children aged 4–11. Our analysis reveals that neonatal hyperbilirubinemia increases the risk of pediatric brain tumors threefold in this age group (Fig. 5n).To further examine the correlation between bilirubin levels and pediatric brain tumor risk across different age groups, in various age groups, we analyzed the Auger et al. dataset^51^, revealing a stronger association, with a 5.3-fold increased risk in children aged 6–11 (**Extended Data Fig. 13j**). Specifically, we observed a higher incidence of tumors in the high-level bilirubin cohort compared to the low-level cohort for MB (**Extended Data Fig. 13k**). Collectively, these epidemiological studies and analyses indicate that elevated bilirubin level is a strong risk factor for brain tumorigenesis in childhood.

## Discussion

Our study provides a comprehensive explanation of the sex differences in MB prevalence and progression between boys and girls, driven by differences in the rate of cerebellar development. At the cellular level, male NPCs are approximately 20 days less differentiated than their female counterparts, which leads to MBs in males exhibiting characteristics—such as incidence and prognosis—that resemble tumors 5.5 years younger than those observed in females. At the molecular level, higher bilirubin levels in the male cerebellum accelerate lipid metabolism, while lower ABCA1 expression slows lipid transfer from microglia. This combination results in slower differentiation of male NPCs, increasing their susceptibility to tumorigenic processes. Elevated bilirubin levels in the male cerebellum appear to be driven by reduced expression of the erythrocyte membrane protein GYPA and enhanced erythrophagocytosis activity in cerebellar microglia. These mechanisms could also extend to other pediatric brain tumors, providing insights for the development of preventive and targeted strategies for children (**Extended Data Fig. 14**).

Our study also explores the origins of these sex differences at the developmental level, highlighting the crosstalk among erythrocytes, microglia, and neural progenitor cells during early embryonic stages. Our scRNA-seq data analysis identifies the common progenitors of these cell types, along with endothelial cells, in the developing mouse brain (**Extended Data Fig. 14c**). This finding is further supported by the developmental trajectory^58,59^. A recent study demonstrates that endothelial cells contribute to the formation of G3 MB from Protogenin-positive (PRTG^+ve^) MYC^high^ NESTIN^low^ stem cells during early development^60^. Furthermore, our results show that sex-specific DEGs in endothelial cells are significantly enriched in neurodevelopmental processes. The findings of this study provide key insights into the fundamental understanding of sex disparities in neurodevelopment and pediatric brain tumorigenesis.

Historically, it has been argued that sex differences in physiological and pathological conditions between males and females are primarily driven by gonadal hormones and sex chromosome-related gene expression^61,62^. However, emerging evidence indicates that some of these differences are mediated by mechanisms beyond the influence of hormonal secretions or X and Y chromosome activity^63–66^. We uncovered how the principal cell types, including erythrocytes, microglia, and NPCs, orchestrate sexual dimorphisms during early brain development. Erythrocytes or RBCs are essential for the survival and health of all vertebrate organisms at every stage of life^67^. Previous studies reported that males have significantly higher RBC count and hemoglobin levels, but increasing susceptibility to various stress, resulting in higher rates of hemolysis^68,69^. We found that male RBCs express lower levels of the GYPA gene, resulting in greater damage to male RBCs compared to female RBCs. These sex-specific characteristics of erythrocytes may drive the differences in microglia-mediated erythrophagocytosis between males and females. These sex differences are further amplified by sex-specific characteristics in microglia, such as lower levels of ABCA1 and higher levels of BLVRB gene expression in males. These factors synergistically limit cellular lipid and cholesterol efflux in male microglia compared to females, ultimately leading to slower NPC maturation. Therefore, our findings provide a comprehensive understanding of the molecular and cellular mechanisms underlying sex differences in neurodevelopment.

Given the absence of known lifestyle-related or environmental risk factors for pediatric brain tumors, there is currently no way to prevent these cancers in children. This study offers hope for preventing, reducing the incidence, and mitigating the progression of MB and other pediatric brain tumors. Our findings suggest that controlling bilirubin levels could potentially reduce the risk of developing pediatric brain tumors by up to fivefold in children aged 6–11 based on the clinical epidemiological datasets^51,52^. A previous case report described a patient with Gilbert syndrome, a genetic condition characterized by elevated bilirubin levels and reduced cholesterol levels, who was diagnosed with MB^70^. Although neonatal jaundice screening before hospital discharge is standard clinical practice, the methods used—such as visual assessments—often lack accuracy and fail to provide systematic data on bilirubin levels in newborns, limiting their utility in predicting the occurrence of postnatal diseases, particularly pediatric brain tumors^71,72^. Our findings in this study provide a foundation for designing epidemiological studies to explore the correlations between neonatal bilirubin levels and the incidence, progression, and metastasis of pediatric brain tumors. These studies will lay the groundwork for future clinical guidelines on infant bilirubin screening and pediatric brain tumor prevention, potentially revolutionizing the current paradigm of pediatric brain tumor diagnosis and treatment.

## Methods

### Cell lines and cell culture

MED8A, D556, D425, the human fetal cerebellar neural progenitor cells (hcNPCs), and 293T were cultured as previously described^16^; The human fetal microglia (HMC3) (CRL-3304) from ATCC were cultured in EMEM with 10% FBS.

### Mouse studies

Animal experiments were performed with the approval of the University of Pittsburgh Animal Care and Use Committee (IACUC) with protocol # 24044987. ICR SCID mice at 4–6 weeks of age were purchased from Taconic Biosciences (Model # ICRS-F/ICRS-M). C57BL/B6 mice at 4–6 weeks of age were purchased from The Jackson Laboratory (Strain # 000664). The presence of a vaginal plug is considered E0.5. Mouse sex was determined through genotyping (The primers are listed in **Supplementary Table 1**). All mice were bred and maintained at CHP Rangos Research Center under pathogen-free conditions with a 12-hour light-dark cycle, a temperature range of 21 to 23°C, relative humidity of 55 ± 10 %.

For MB models, adult ICR SCID or C57BL/B6 mice were anesthetized and injected with cells (1 × 10^5^) as previously described^16^. For virus-induced spontaneous MB models, postnatal C57BL/B6 mice were anesthetized on ice, then placed into stereotactic apparatus equipped with z-axis. Lentiviruses were injected into 1 mm deep of the cerebellum vermis on midline. Following injection, mice were warmed on a heating pad and monitored to ensure recovery. Animals were monitored for tumor development by assessing neurological functions/signs (e.g., hunchback, seizure, and posterior paralysis). In vivo MRI with contrast brain image was carried out using a Bruker BioSpec 70/30 USR spectrometer operating at 7-Tesla field strength with the parameters: FOV 3.0 cm × 2.0 cm, acquisition matrix 384 × 256, acquisition slice thickness 0.60 mm, TR/TE = 2177/14 ms. Mice with neurological deficits or moribund appearance were sacrificed. Brains were removed after transcardial perfusion with 20 ml ice-cold PBS and 20 ml 4% paraformaldehyde (PFA), then fixed in 4% PFA for paraffin-embedded or OCT-frozen tissue blocks.

The glioma mouse model was obtained from Dr. Jian Hu (UT MD Anderson Cancer Center, Houston, TX) and was maintained and induced with tamoxifen at P7 as previously described^73^.

For mouse treatment, PLX5622 (MEDCHEMEXPRESS LLC, HY-114153) was delivered at a dose of 45 mg/kg body weight through oral gavage into the stomach of the pregnant mouse using curved feeding needles (Kent Scientific Corporation, FNC-20–1.5–2) from E6.5 to E18.5. Probucol (Thermo Scientific, AC469490050) was delivered at a dose of 100 mg/kg body weight through IP injection every two days from P0 to P7. PHZ hydrochloride (Sigma-Aldrich, 114715) was delivered at a dose of 100 mg/kg body weight through IP injection every two days from P0 to P7.

### Lentivirus production and transduction of target cells

The expression vectors were generated by cloning the respective open reading frame into the lentiviral vector through Golden Gate reaction using Aar I for seamless cloning. The lentiviral CRISPR/Cas9 vectors were generated by ligating the oligos of sgRNA sequences (**Supplementary Table 2**) into lentiCRISPRv2-Blast (Addgene, 83480) and then validated by Sanger DNA sequencing. Gene expression in lentivirus-infected target cells was validated by RT-qPCR (The primers are listed in **Supplementary Table 3**). Lentiviruses were produced in 293T cells with a packaging system as previously described^16^.

### Hematoxylin and Eosin (H&E), Immunohistochemistry (IHC), Immunofluorescence (IF), and Immunocytochemistry (HCC)

For H&E staining, brain sections were stained per the vendor’s (HAE-1-IFU) instruction. Briefly, the slides were incubated in hematoxylin for 3 minutes and incubated in bluing reagent for 15 seconds. Slides were counterstained with Eosin Y for 1 minute and subsequently dehydrated prior to mounting. For IHC staining, brain sections were stained as previously described^16^. The images were acquired by Nikon Eclipse E800 microscopes. For IF staining, mouse tissues were processed for OCT frozen blocks via a sucrose gradient. OCT frozen sections were cut at 10 μm and subsequently thawed at room temperature for 30 min, rinsed, and rehydrated with phosphate-buffered saline (PBS) 3 times. Slides were fixed at room temperature for 20 minutes using 4% PFA, then washed 3 times with PBS. Antigen retrieval was performed using 100x Antigen Unmasking Solution (Vector Laboratories, H-3300-250) and boiled for one hour, followed by cooling and two PBS washes. Slides were blocked with Normal Horse Serum 2.5% (Vector Laboratories, S-2012-50) at room temperature for one hour. The sections were incubated with indicated primary antibodies overnight at 4°C followed by species-appropriate secondary antibodies coupled to AlexaFluor dyes (488, 594, 647, Invitrogen) for 1 hour at room temperature. Mounting Medium with DAPI (Abcam, ab104139) was used to mount coverslips. The images were acquired with a Leica STELLARIS 5 confocal microscope or Leica DMI8 microscope and analyzed by ImageJ and Photoshop. The information about antibodies used for these assays are described in **Supplementary Table 4.**

### Flow cytometry and FACS sorting

Mouse blood was collected, and erythrocytes were lysed in ACK lysing buffer (Gibco, A1049201) if need. Cerebellum, cerebrum, other brain regions, spleen and liver were harvested following vascular perfusion with 20 ml sterile ice-cold PBS. Organs were digested in 3.2 mg/ml collagenase IV (Worthington Biochemical Corporation, LS004209), 1 mg/ml Deoxyribonuclease I (Worthington Biochemical Corporation, LS002007) and 2 mg/ml soybean trypsin inhibitor (Worthington Biochemical Corporation, LS003587) in DPBS for 10 min at 37 °C while shaking to separate cells. The cells were analyzed in a BD Fortessa or Cytek Aurora analyzer following incubation with BODIPY, CD45, CD11b, P2ry12, CD133, Ter-119, GYPA, or Ghost red 780 (Cytek, 13-0865-T100) for 30 minutes on ice. For any staining containing BODIPY (Invitrogen, D3922) or BODIPY^CE^ (Invitrogen, C12680), the wash buffer is DPBS. For other staining, the wash buffer is PBS containing 0.5% bovine serum albumin and 2mM ethylenediaminetetraacetic acid (EDTA). Cells were washed twice in indicated wash buffer and resuspended in 200 uL of buffer for flow cytometry. FACS sorting was performed using the BD FACSAria cell sorter. Data were analyzed using FlowJo software (v.10.7.1). The information about antibodies used for these assays are described in **Supplementary Table 4.**

### RNA isolation, RT-qPCR, RNA-seq, and *scRNA-seq*

RNA was isolated with RNeasy Plus Mini Kit (QIAGEN, 74134) and then used for first-strand cDNA synthesis (Invitrogen, 28025-013) or (Vazyme, R302). RT–qPCR was performed using PowerUp SYBR Green Master Mix (Applied Biosystems, A25742) and the Taq Pro Universal SYBR qPCR Master Mix (Vazyme, Q712); the relative expression of genes was normalized using ribosomal protein L39 (*RPL39*) or *ACTB* as a housekeeping gene.

For RNAseq, sequencing libraries were generated using NEBNext Ultra RNA Library Prep Kit for Illumina following the manufacturer’s recommendations and were sequenced as previously described^16^. Reads were aligned using Hisat2 (v.2.1.0) against the hg38 or mm10 genome and transcriptome. The statistical environment R was used to perform all the statistical analysis and graph plots.

For scRNA-seq, mouse cerebellum and tumor were dissociated as described above. Dissociated single cells were checked for viability using a ViaStain AOPI staining solution (Nexcelcom Biosciences, CS2-0106-5ML) and counted using a Cellometer Auto 2000 Viability Counter (Nexcelcom Biosciences). Any dead cells were removed afterwards using a Miltenyi Biotech Dead Cell Removal kit (130-090-101). CDNA libraries were prepared using the Chromium Next GEM Single Cell 3’ Reagent Kit v3.1 (10x Genomics). For quality control, the libraries were tested using High Sensitivity D5000 ScreenTape and 4200 Tapestation system (Agilent Technologies) and sequenced using a 10B lane of Novaseq X Plus (Illumina). For scRNAseq analysis, genes not expressed in any of the cells had already been removed. Cells with less than 200 genes or more than 5, 000 genes expression or more than 15% mitochondrial genes expression were removed using Seurat (v.3.2.3). Using known marker genes, clusters by UMAP were assigned to cell types. Pseudotime analysis was performed with monocle3 (v.1.0.0).

### Coculture, condition medium culture and soft agar assay

For coculture, equal number of HMC3 and hcNPCs were seeded on plastic plates. Cells were cultured in the hcNPC medium as previously above. For conditioned medium culture, HMC3 were cultured in the hcNPC medium for 3 days. The medium was centrifuged at 2000 rpm for 10 minutes and the supernatant was collected to culture the hcNPCs. The hcNPCs were overexpressed with MYC^S62D^ lentivirus overnight and equal numbers were utilized for soft agar assay as previously described^74^. The fluorescence and bright-filed images were acquired with an ECHO Revolve, and the scanning images were acquired with Epson Perfection V800.

### Cholesterol transfer assay

Microglia were cultured in the B27 depleted hcNPC medium with 2 μM BODIPY^CE^ for 12 hours to label cholesterol. After removing BODIPY^CE^ by washing with DPBS, the HMC3 cells were cultured in the B27 depleted hcNPC medium for 3 days. The 3-day cultured medium was collected as condition medium and was used to culture hcNPCs. The BODIPY^CE^ MFI of cultured hcNPCs was measured by flow cytometry.

### Bilirubin treatment

Microglia were cultured in the hcNSC medium with indicated concentrations of bilirubin. The cultured medium was collected after 3 days and used for hcNPC culturing as before. The microglia were stained with BODIPY for 30 minutes after culturing. The BODIPY MFI of collected microglia was measured by flow cytometry as described above.

### Erythrocyte preparation, PKH26 labeling and injection

Whole blood collected from C57BL/6J WT mice was centrifuged at 400g for 10 min. The pellet was resuspended in DPBS and heated for 20 min at 48 °C under continuous shaking at 900 rpm, generating stressed erythrocytes as described^75^. The stressed erythrocytes were labeled with PKH26 (Sigma-Aldrich, MINI26-1KT) following the manufacturer’s instruction. 0.5×10^9^ PKH26 labeled stressed erythrocytes were injected into the temporal or facial vein of the P0 pups under macroscope. Peripheral blood was collected at differing times labeled and measured using flow cytometry.

### Meta-analysis

Literatures were searched in PubMed for these search terms: (neonat* OR newborn* OR infant*) AND (bilirubin OR phototherapy OR hyperbilirubinemia OR jaundice) AND (cancer or malign * OR tumor* or neoplasm*). Data and studies containing brain tumor samples were collected for further analysis. For studies with overlapping samples, the most recent study was retained. Common effect model was used when the inconsistency index statistic I^2^ is lower than 30%. Random effects model was used when the I^2^ is larger than 30%. Risk ratio with 95% CIs was calculated with the Mantel-Haenszel method. Meta (v.7.0.0) package was used for analysis.

### Spatial transcriptome

Spatial orientation of cells chosen for Neighborhood Enrichment Analysis from MouseBrain_P7_section1_singlecell (Stomics dataset STDS0000139). First, a subset containing only the cerebellar region of the full dataset was obtained. Next, Seurat was used to perform the standard cluster analysis workflow and name the cell clusters using the expression profiles of common cerebellar cell type markers. For the neighborhood analysis, python package Squidpy (v1.6.0) was used to calculate an enrichment score based on proximity on the connectivity graph of cell clusters. The number of observed events is compared against N=1000 permutations and a z-score are computed.

### Clinical information

The pathology analysis of MB and glioma samples was conducted by at least two experienced neuropathologists. Clinical information from Xiangya Hospital were used and analyzed with approval by the institutional review board (number 202310205). The study was compliant with all ethical regulations.

### Statistics analysis and reproducibility

All the boxplots display the interquartile range (IQR), whiskers denote quartile 3 + 1.5 × IQR or quartile 1 – 1.5 × IQR. Data points outside the whiskers are considered outliers. Column bar plots show the mean with SD. Data were judged to be statistically significant when P < 0.05. Statistical parameters, including the exact value of n, the definition of center, dispersion, precision measures, statistical tests, and statistical significance were reported in the Figures and Figure Legends. Sample sizes were not predetermined using statistical methods. The investigators were blinded to assess protein expression in IHC/IF experiments; other data collection and analysis were not performed in a blinded manner. No animals or data were excluded except for overlapping samples in the MB studies, low-quality cells during scRNAseq analysis, and studies with overlapping samples in meta-analysis. AUCell (v.1.8.0) and G3 features from our previous study^16^ were used to calculate the G3 score for scRNA-seq data, and GSVA (v.1.36.3) and other pathway features were used to calculate the other indicated pathway score for bulk RNA-seq data. R (v.3.5.1) and GraphPad Prism (v.10.3.1) were used for statistical analyses.

## Supplementary Material

Supplementary Files

This is a list of supplementary files associated with this preprint. Click to download.

- SupplementaryTables.xlsx
- Zouetal.ExtendedFigures.pdf

## Data Availability

The RNA-seq and scRNA-seq data that were generated for this study have been deposited in the Gene Expression Omnibus (GSE292964, GSE292965). Previously published data that were re-analyzed here are available: the mouse scRNA-seq datasets include GSE118068, GSE122357, GSE165371, GSE209915, GSE209917, PRJEB23051, PRJNA637987, SRP135960, GSE165371, GSE119945, GSE186068, GSE186069, GSE100597, GSE109071, and E-MTAB-6967, the human scRNA-seq datasets include the Human Cell Atlas (https://www.covid19cellatlas.org/aldinger20), GSE156793, the Neuroscience Multi-omic (NeMO) Archive (RRID:SCR_002001), GSE134355, GSE165371, GSE127774, and GSE157329, the human expression/methylation profiles and clinical information are from GSE124814, GSE10327, GSE148389, GSE50765, GSE68956, GSE85217, Children’s Brain Tumor Network, E-MTAB-10767, cBioPortal (mbl_broad_2012 and mbl_icgc), Dr. Thompson^76^, Dr. Huybrechts^77^, and Dr. M. D. Taylor, the mouse tissue expression profiles are from GSE100421, GSE114760, GSE173888, GSE64425, GSE84462, GSE147178, GSE137633, GSE188816, GSE104633, GSE112772, GSE164311, GSE183901, GSE60415, and GSE98299, the human cerebellum expression profiles and clinical information are from GSE167447, GSE22569, GSE25219, and GSE44971, and the P7 mouse cerebellum spatial transcriptomic profiles are from Stomics dataset (STDS0000139). All other data supporting the findings of this study are available from the corresponding author on reasonable request.
